# The role of reactive oxygen intermediates in experimental coccidioidomycois in mice

**DOI:** 10.1186/1471-2180-11-71

**Published:** 2011-04-11

**Authors:** David A Margolis, Suganya Viriyakosol, Joshua Fierer, Theo N Kirkland

**Affiliations:** 1 Veterans Affairs San Diego Healthcare System (111F) 3350 La Jolla Village Dr, San Diego, CA 92161, USA; 2 Veterans Medical Research Foundation, 3350 La Jolla Village Dr. San Diego, CA 92161, USA; 3 Department of Pathology University of California, San Diego, San Diego, California, 92093, USA; 4 Department of Medicine, University of California, San Diego, San Diego, California, 92093 USA; 5 GlaxoSmithKline Infectious Diseases Medicine Development Center 5 Moore Drive Research Triangle Park, NC 27709 USA

**Keywords:** Coccidioidomycosis, host response, oxidative burst

## Abstract

**Background:**

Coccidioidomycosis is usually a self-limited infection in immunocompentent people. In immunocompentent human beings second infections due to *Coccidioides *are very rare, indicating that recovery from infection results in protective immunity. In experimental animals, immunization with several different proteins or attenuated mutants protects against a virulent challenge. To explore what mechanisms are responsible for protective immunity, we investigated the course of *Coccidioides *infection in the gp91^phox ^knock out mouse that has a defect in the oxidative burst that results in chronic granulomatous disease.

**Results:**

We found that the gp91^phox ^knock out mice were somewhat more resistant to intraperitoneal infection and equally as resistant to low dose intranasal infection, but slightly more susceptible to high dose intranasal infection compared to control mice. The gp91^phox ^knock out mice made a more robust inflammatory response to infection than controls, as measured by histology and production of inflammatory cytokines. The gp91^phox ^knock out mice were as protected by immunization with the recombinant *Coccidioides *protein Ag2/PRA as the controls were against either intraperitoneal or intranasal infection. *Coccidioides immitis *arthroconidia and spherules were significantly more resistant to H_2_O_2 _treatment in vitro than *Aspergillus fumigatus *spores.

**Conclusion:**

These data suggest that oxidative burst may not be required for protective immunity to coccidioidomycois.

## Background

*Coccidioides immitis *and *posadasii *are pathogenic fungi that grow in the arid soils of the southwestern United States, Mexico and Central and South America. Mycelia in the soil give rise to infectious arthroconidia, which, when aerosolized, can be inhaled. The severity of coccidioidomycois (Valley Fever) ranges from a mild self-limited disease to a severe pneumonia and widely disseminated infection requiring lifelong antifungal therapy [1]. The risk factors for the more severe forms of disease include ethnic background (Filipino, African-American, Hispanic), male sex, increasing age, pregnancy and immunosuppression (HIV, malignancy, organ transplantation) [2-4].

The role of polymorphonuclear leukocytes (PMNs) macrophages and the oxidative burst in the defense against *Coccidioides *is not clearly defined. PMN's are the first cell to respond to inhaled arthroconidia [5]. Although arthroconidia are sensitive to products of the oxidative burst [6,7] and are phagocytosed by PMNs [8-10], fewer than 20% of arthroconidia are killed by human PMNs [8,9,11,7]. Once arthroconidia mature into spherules they become resistant to phagocytosis and PMN killing because of their size (60-80 microns) [9,12]. The extracellular matrix of spherules also appears to resist attachment by PMNs [9]. Rupture of spherules releases endospores that have been shown to activate the oxidative burst and are readily phagocytosed by PMN's [9,11]. In spite of this, endospores appear to be resistant to killing by PMNs [9,11]. There has not been an adequate study of *Coccidioides *in a neutropenic infection model, to understand the importance of neutrophils and macrophages on disease progression.

Coccidioidomycois is usually a self-limited infection. In immunocompentent people pulmonary infections resolve without drug treatment greater than 95% of the time [1]. In addition, human infection leads to protective immunity and some types of immunization have proven protective in mice [13-17]. We have found that the protective immunity to antigen 2/proline rich antigen (Ag2/PRA) in mice requires MHC-Class II-dependent CD4 cells but did not require CD8 T-cells [18]. IL-12 is also required, suggesting that a Th1 immune response was important for protective immunity [18]. Mice lacking interferon-γ were not protected by immunization with Ag2/PRA [18]. One issue these studies did not address was what type of effector mechanism was responsible for actually killing the fungus or inhibiting its growth. Because reactive oxygen intermediates are so important for natural resistance to *Aspergillus *species, we asked what role this mechanism plays in natural and acquired resistance to coccidioidomycosis using the gp91^phox ^knock out (KO) mouse.

To address the role of the oxidative burst, we used C56Bl/6 mice with a deletion in the NADPH oxidase gene gp91. These mice were developed in 1995 by Pollack as a chronic granulomatous disease (CGD) mouse model [19]. This mouse is characterized by functionally defective PMNs and macrophages because of a mutation in NADPH oxidase in the X-linked gene *gp91*^*phox *^(where phox stands for phagocyte oxidase). This gene encodes a 91 kD subunit of the oxidase cytochrome b. These mice have increased susceptibility to *Aspergillus *and *Staphylococcus aureus *infection because of ineffective oxidative killing by their PMNs. In this study we analyze the response of the gp91^phox ^KO mice to infection with *Coccidioides immitis *and evaluate the response of these mice to immunization.

## Methods

### Mice

B6.129S6-*Cybb*^*tm1Din*^/J (referred to as gp91^phox ^KO) mouse breeding pairs were obtained from Jackson Laboratory (Bar Harbor, ME) and bred in a specific pathogen free environment. Both male and female mice express the gp91^phox ^mutation. 6-12 week old female mice were used for all experiments. C57Bl/6J female (B6) mice 6-12 week old mice were used as controls. The Subcommittee on Animal Studies approved all experimental protocols involving animals.

### Fungus

The R.S. strain of *C. immitis *was used as the challenge strain. Cultures of mycelia were harvested after 60 days. Arthroconidia were prepared from mycelia as previously described [13].

### Immunization and infection

Mice were immunized with 2 μg Ag2/PRA [14] (a gift of Dr. John Galgiani) and 10 μg of CpG oligonucleotide [18] in a 50/50 emulsion of saline and mineral oil, injected in a total volume of 0.2 ml subcutaneously. Non-immune controls were injected with 0.2 ml of a 50/50 emulsion of saline and mineral oil subcutaneously. The immunization or control injection was repeated 14 days later. 14 days later (28 days after the first immunization) the mice were challenged with 150 R.S arthroconidia in 0.5 ml saline into the intraperitoneal space (I.P.). 14 days after the challenge the mice were euthanized. The left lung was removed, homogenized in 2 ml saline, serially diluted, and quantitative culture done.

Pulmonary infection was initiated with 150 or 250 arthroconidia intranasally in 20 μl saline after mice were anesthetized with ketamine and xylazine (0.1 ml of a cocktail containing ketamine (15 mg/ml), xylazine (16 mg/ml) in saline was injected i.p). After infection, they were rested on a heating pad and monitored until they woke up in about 1 h. The mice were monitored for mortality for 30 days.

### Real-time Quantitative PCR for Lung Cytokines

Groups of 4 mice were infected with 150 arthroconidia I.P. Twelve days after infection the upper lobe of the right lung of a mouse was removed into 2 ml Ultraspec (Biotecx) and immediately homogenized. Total RNA was extracted as described in the manufacturer's protocol. RNA was quantified and analyzed for integrity using a Bioanalyzer (Biorad Experion). cDNA was synthesized using superscript VILO cDNA synthesis kit (Invitrogen). Taqman gene-specific primer/probes for mouse cytokines and 18S were purchased from Applied Biosystems. The real-time quantitative PCR reactions and data analysis were carried out by UCSD CFAR genomic core according to the manufacturer's protocol using an ABI Prism 7900 HT sequence detection system. Amplification of 18S RNA was performed to standardize the amount of sample added to each reaction.

### Susceptibility to Oxidative Stress

*Aspergillus fumigatus *spores were harvested from mature slants in distilled water. *C. immitis *arthroconidia were harvested from mycelia by beating with glass beads as previously described [13]. *C. immitis *spherules were grown in modified Converse media for 7 days as previously described [20]. About 200 organisms were incubated with various concentrations of H_2_O_2 _in 1 ml saline for 45 minutes at room temperature. The fungi were collected by centrifugation, washed in saline by centrifugation and the sediment cultured on glucose yeast extract agar. The number of colonies was counted and compared to a control that was processed as above but not treated with H_2_O_2. _Each experimental point was determined in triplicate; the mean and S.E.M. is plotted.

### Statistics

All quantitative culture data and quantitative mRNA data was compared using the Mann-Whitney U test. Survival data was analyzed by the Kaplan-Meier test. Both tests were done with Prism GraphPad 4 software (San Diego, CA).

## Results

**Figure 1:**
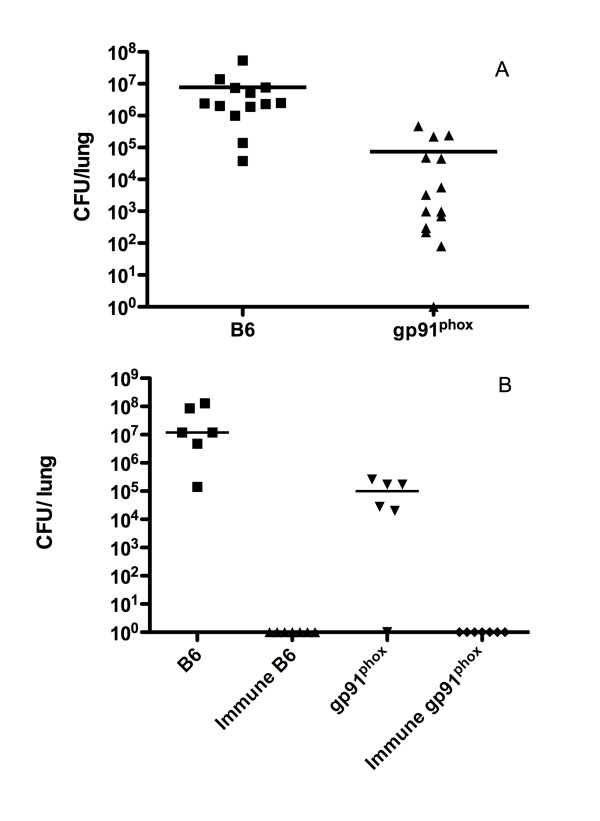
**The number of CFU of *Coccidioides *found in the lungs of gp91**^**phox **^**KO and B6 controls 14 days after intraperitoneal infection**. Each symbol represents a mouse; the line represents the median. Panel A: non-immune mice. Panel B: Immune and non-immune mice of the two strains are compared.

We initially tested the innate resistance of gp91^phox ^KO mice to intraperitoneal infection with *C. immitis*. The number of CFU/lung was determined by quantitative culture on day 14. Figure 1A shows the results. The gp91^phox ^KO mice had slightly lower numbers of CFU/lung compared to the B6 controls (p < 0.001, Mann-Whitney U). We then compared the innate and acquired resistance of C57Bl/6 mice and the gp91^phox ^KO mice to intraperitoneal challenge with *C. immitis*. Animals were immunized with Ag2/PRA as described in Methods. They, and non-immune controls were challenged with 150 arthroconidia I.P. and sacrificed 14 days later. The number of CFU/lung was determined by quantitative culture (Figure 1B). Once again the number of CFU/lung was slightly lower in the unimmunized phox KO mice compared to C57Bl/6 controls. More striking was the observation that both types of mice were completely protected by immunization.

**Figure 2:**
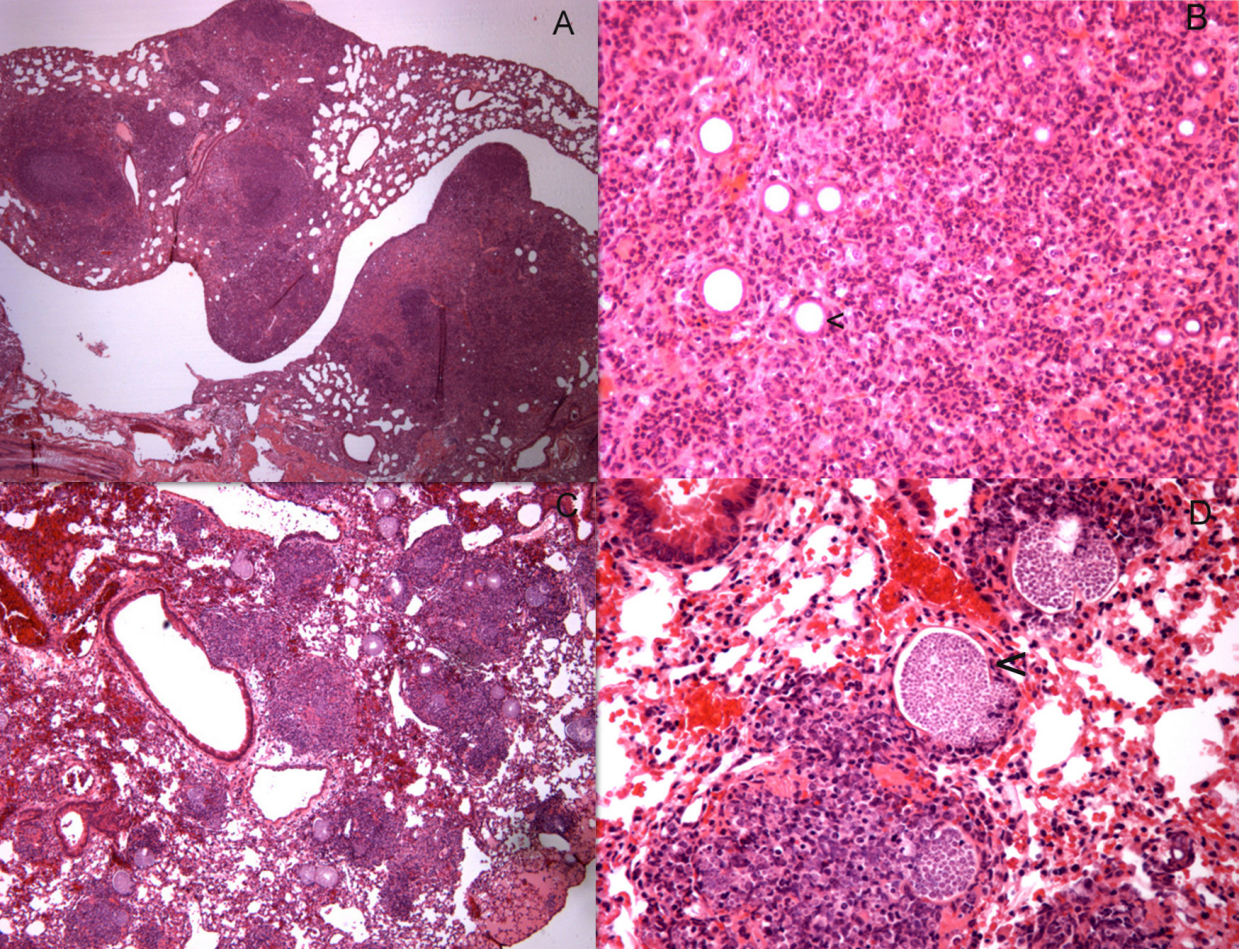
**Hematoxylin and eosin stained sections of lungs from gp91**^**phox **^**KO mice (panels A and B) and B6 mice (panels C and D) 14 days after intraperitoneal infection**. Panels A and C: 2X magnification: panels B and D: 40X magnification. The arrowheads in panel B and D indicate spherules.

Representative images of the histological evaluation of the infected lungs in non-immune B6 and gp91^phox ^KO mice are shown in Figure 2. The most striking difference is that the B6 mouse lungs contain more mature spherules than the gp91^phox ^KO mouse lungs do, as would be expected from the quantitative culture data. In both mouse strains the predominant cellular response is neutrophilic. The inflammatory foci are larger in the gp91^phox ^mice than in the controls, despite the smaller number of spherules found in these lesions.

**Figure 3:**
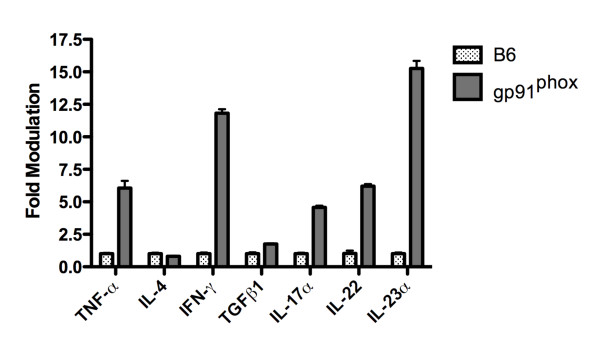
**The amount of mRNA for the indicated cytokines found in the lungs of gp91**^**phox **^**KO and B6 control mice 14 days after intraperitoneal infection**. The bars represent the mean and the error bars the standard deviation. The amount of each of the cytokines in the uninfected B6 mice was set at 1.

We also measured the amount of mRNA coding for selected cytokines in the lungs of B6 and gp91^phox ^KO mice infected with *Coccidioides *(Figure 3). We found that the infected gp91^phox ^KO mice expressed higher mRNA levels for all the cytokines tested compared to the B6 mice, except for IL-4 and TGF-β1. The most striking differences between the levels of mRNA in the gp91^phox ^KO and B6 mice were in TNF-α (p = 0.012), interferon-γ (p = 0.008), IL-17α (p = 0.002), IL-22 (p = 0.003) and IL-23 (p = 0.002).

**Figure 4:**
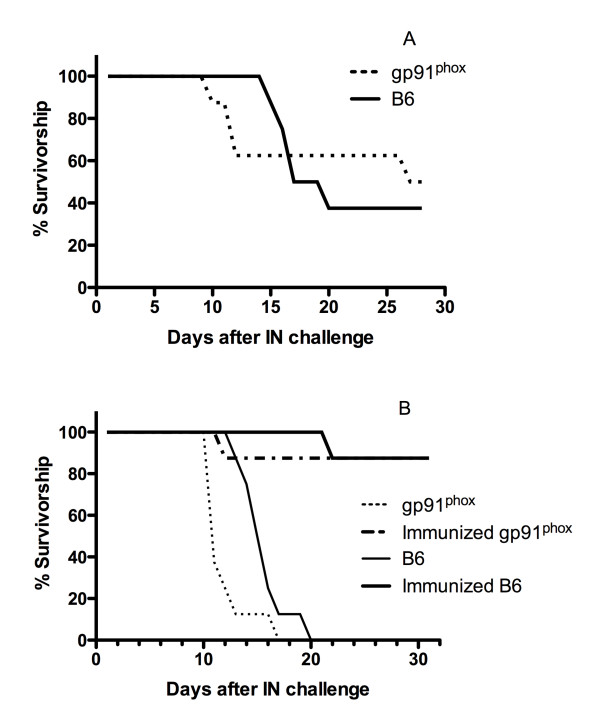
**Survival of groups of 8 gp91**^**phox **^**KO and B6 mice after intranasal infection with 150 (Panel A) or 250 (Panel B) arthroconidia**. In panel B immune and non-immune mice are compared.

We wanted to compare the gp91^phox ^KO and control mice in the more physiologic intranasal model of infection. In this model mortality is used as the endpoint rather than quantitative culture. C57Bl/6 and gp91^phox ^KO mice were infected with 150 arthroconidia intranasally and observed for 30 days. The mortality curves are shown in Figure 4A. Both control and gp91^phox ^mice died at the same rate after intranasal infection. To determine whether the gp91^phox ^KO mice could be successfully immunized in this model of infection, we immunized the two strains of mice as described in Materials and Methods and challenged them intranasally with 250 arthroconidia. The immune mice were compared to non-immune controls, which had been given adjuvant only. The survival curves are shown in Figure 4B. With the larger challenge all the non-immune mice died by day 20 and the gp91^phox ^KO mice died slightly more quickly than the B6 mice (p = 0.023). In contrast, 7 of 8 immune B6 and gp91^phox ^KO mice survived for 31 days (p < 0.001 for both B6 and gp91^phox ^compared to non-immune control). There was no difference in survival between the immune B6 and gp91^phox ^KO mice (p = 0.715).

**Figure 5:**
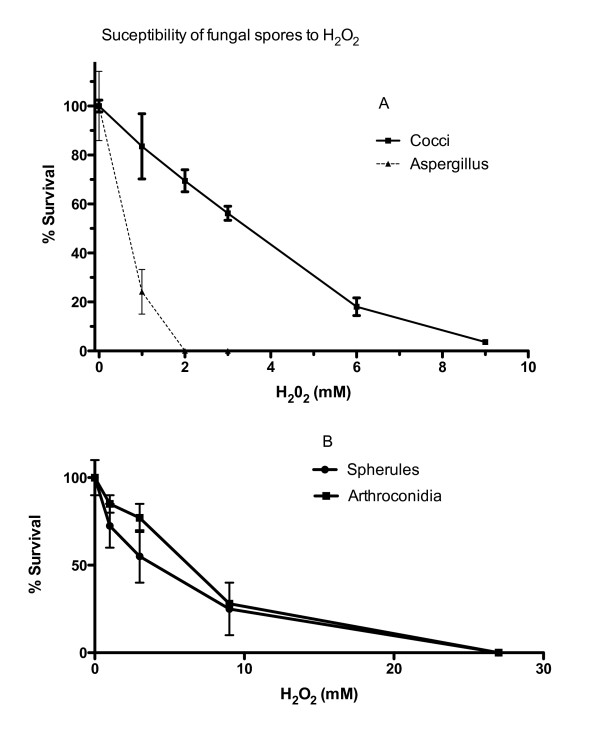
**The susceptibility of fungal spores to H2O2.** A: Survival of *C. immitis *arthroconidia and *A. fumigatus *spores after 45 minutes exposure to the indicated concentrations of H_2_O_2_. B: Survival of *C. immitis *arthroconidia and spherules to 45 minutes exposure to the indicated concentrations of H_2_O_2_. In both cases the mean and S.E.M. is plotted.

We compared the lethal effect of H_2_O_2 _on *Aspergillus fumigatus *spores and *C. immitis *arthroconidia and spherules. The results are shown in Figure 5. Clearly, the arthroconidia require at least five times higher concentrations of H_2_O_2 _to kill them compared to *Aspergillus fumigatus *spores. Similar results were seen in three independent experiments. There was no difference in the susceptibility of spherules and arthroconidia to H_2_O_2 _(Figure 5B). We did not test susceptibility to any other reactive oxygen species.

## Discussion

The objective of this study is to determine what effect the deletion of gp91 phox had on the innate and acquired immune response to *Coccidioides*. We examined the responses to two different routes of infection: intraperitoneal, which is not physiologic but has quantitative culture as an endpoint, and intranasal resulting in primary rather than hematogenous pulmonary infections. In the latter model mortality was the endpoint. Although intraperitoneal infection is not the physiologic natural route of infection, the studies done so far in many laboratories have not identified any major differences in the immunological protective mechanisms required for coping with intraperitoneal versus intranasal infection. In both circumstances T-cell mediated immunity is required and a Th-1 immune response is important [18,21,22]. Furthermore, the genetics of resistance to Coccidioides infection by these two routes is very similar (unpublished observation) [10,23]. The only significant difference documented between the two routes of infection so far is that some vaccines that are protective in the intraperitoneal model are not protective in the intranasal model [24]. C57BL/6 mice are extremely susceptible to intranasal infection with *Coccidioides *so that very small difference in inoculum can have major effects on mortality rate. We have found that the intraperitoneal route of infection is more reproducible and predictable, so we chose to do preliminary experiments using this model. In both these infection models, the gp91 phox mutation had no effect on acquired immunity to Ag2/PRA. These data suggest that reactive oxygen intermediates may not be required for protective immunity.

The situation in non-immune mice is less clear. In the intraperitoneal model of infection, the gp91^phox ^KO mice had significantly fewer organisms in their lungs compared to the controls. This may be due to the more exuberant inflammatory response seen in the gp91^phox ^KO mice compared to the B6, as measured by histology and amount of Th1 and Th17 cytokine mRNA in the infected lung. In the intranasal model of infection, no difference between the gp91^phox ^KO and B6 was seen when the mice were challenged with 150 arthroconidia, but there was a small difference in survival between the two mouse strains when they were challenged with a larger number of organisms. The increased mortality rate may also be due to a more vigorous inflammatory response in the gp91^phox ^KO mice.

We also found that *C. immitis *arthroconidia and spherules were significantly more resistant to killing by H_2_O_2 _*than Aspergillus fumigatus *spores. gp91^phox ^KO mice are susceptible to pulmonary *Aspergillus *infection, so this is a potential explanation for the difference in susceptibility of the gp91^phox ^KO to these two fungi. However, since it is not clear that ROI kill fungi directly (see below) the significance of this observation is not clear.

More studies in CGD mice have been done with the gp47^phox ^KO rather than the gp91^phox ^KO. Mice with both mutations have the CGD phenotype but there may be differences between the two. The observation that gp47^phox ^KO and gp91^phox ^KO mice make a more robust inflammatory response than control mice with an intact respiratory burst has been previously made in mice experimentally infected with *Aspergillus fumigatus *[25] or in mice given intra-tracheal zymosan [26,27]. The mechanism of this exaggerated inflammatory response to *Aspergillus fumigatus *infection was thought to be a defect in a superoxide dependent step in tryptophan metabolism [26]. The exaggerated response to zymosan in gp47^phox ^mice was thought to be due to a failure to activate Nrf2, a redox-sensitive anti-inflammatory regulator [26].

The mechanism by which phagocytes inhibit and damage fungi is complex. NADPH oxidase is not required for phagocyte killing of *Aspergillus fumigatus *conidia [28,29]. This growth phase of *Aspergillus fumigatus *is inhibited by lactoferrin-mediated iron depletion [28]. In contrast, inhibition of the hyphal form of *Aspergillus fumigatus *requires NADPH oxidase [28,30]. *Aspergillus nidulans *lacking the catalase genes are capable of causing disease in gp47^phox ^KO mice, which suggested that reactive oxygen intermediates might not be inhibiting the organism directly [30]. It has been suggested that activation of intracellular proteases by reactive oxygen intermediates is important for killing *Candida *and several types of bacteria [31]. There is one report that administration of pentraxin 3 protected gp47^phox ^mice from experimental *Aspergillus fumigatus *infection, suggesting that this molecule in important for resistance to *Aspergillus fumigatus *and may be lacking in CGD mice [32].

The only evidence that primary pathogenic fungi are more virulent in CGD mice is a study with *Sporothrix schenckii *[33]. These investigators found that gp91^phox ^KO mice infected with *Sporothrix schenckii *intradermally died within three months, whereas control mice survived this infection. They also found that PMN from gp91^phox ^KO mice were not able to control the growth of *Sporothrix schenckii *as well as the controls. We have not been able to find any published data on *Blastomyces dermatitidis*, *C. immitis *or *Histoplasma capsulatum *experimental infections in CGD mice.

People with chronic granulomatous disease have increased susceptibility to Aspergillus infections and, to a lesser extent, infections due to other opportunistic fungi [34]. There have been no reports of increased susceptibility to the primary pathogenic fungi *Coccidioides*, *Histoplasma capsulatum*, *Blastomyces dermatitidis *or *Sporothrix schenckii*. One expert states that these infections are not a problem in chronic granulomatous disease [34]. One CGD patient has been observed to recover uneventfully from pulmonary coccidioidomycosis without anti-fungal therapy (J. Galgiani, personal communication).

The observation that NADPH oxidase is not required for a protective immune response to experimental coccidioidomycosis raises the question of what immune mechanisms used to kill spherules and endospores *in vivo*. One potential protective immune effector mechanism is oxidative stress due to nitric oxide. We have previously reported that IL-10 exacerbates the course of experimental coccidioidomycois and inhibits nitric oxide synthase [35]. On the other hand, a very recent study suggests that *Coccidioides *is resistant to killing by NO and that mice with a deletion mutation in inducible nitric oxide synthase are able to kill *Coccidioides *[36]. *Coccidioides *spherules can be very large (more than 60 μM in diameter) and therefore difficult to phagocytose. Perhaps inhibiting the growth of the endospore controls the growth of the organism. Understanding the mechanisms of protective immunity is important for optimally preventing and treating infections with this pathogenic fungus. These finding may also be relevant for other primary pathogenic fungi, such as *Histoplasma capsulatum *and *Paracoccidioides brasiliensis*.

## Conclusions

Mice with the CGD phenotype are not more susceptible to *Coccidioides immitis *infection and they are completely protected by effective immunization. This suggests that some mechanism other than reactive oxygen intermediates may be responsible for protective immunity.

## List of Abbreviations

B6: C57Bl/6; CGD: Chronic granulomatous disease; I.P.: intraperitoneal; ROI: Reactive oxygen intermediates; TNF: α - Tumor necrosis factor-α.

## Authors' contributions

DM performed many of the experiments and participated in writing the manuscript; SV performed many of the experiments and participated in writing the manuscript; JF participated in writing the manuscript; TK supervised the work and wrote the manuscript. All authors read and approved the final manuscript.
